# A Case of Leio-Myom of the Vagina and Uterus

**Published:** 1885-08

**Authors:** Henry T. Byford

**Affiliations:** 125 State Street; Chicago


### REPORT OF CASE OF LEIO-MYOM OF VAGINA AND UTERUS. *By* HENRY T. BYFORD, M. D., *Chicago.*

(Read before the Chicago Gynecological Society, 19th of June, 1885).

The patient was a widow, about 35 years old. She was married ten years without becoming pregnant. She had no decided symptoms of disease except an occasional backache and some leucorrhœa, and frequent menstruation. She was treated for uterine inflammation a short time previous to her husband's death, three years ago, without any tumor being discovered. Since then she has menstruated every two weeks and gives over-exertion at the time of his death as the cause. The flow lasted five days and each time nearly the same in quantity as the monthly flow always had been. Twelve years ago she noticed a tumor about the size of a hickory nut just inside of the vagina. This had since been steadily growing, until its protrusion from the vagina, about Jan. 12th, '85, and had caused her no trouble. Even at that time the pain was promptly relieved by two or three doses of an opiate administered by Dr. Doering, who first saw her. Within two or three days, a black spot appeared upon its distal end. In a week's time the whole tumor was swollen, black and emitted a gangrenous odor. Being unable to persuade her to have the growth removed, I cauterized it every two days with strong carbolic or nitric acid, and surrounded it with cotton dipped in carbolated oil, but I did not succeed in preventing slight septicæmia. As the capsule sloughed off, and left healthy tissue underneath, I attempted to cut off the tumor gradually, by silver wire drawn tightly about its base, twisted as tightly each day as she could easily bear. After this cutting half way through, I found that the parts had united behind the advancing wire. Having thus accomplished no good except to contract the whole pedicle from about two inches to a little over one inch in diameter, I removed the wire and refused to do anything more for her until she consented to submit to an operation for its removal. A few days later I slowly crushed the tumor off under anæsthesia, and applied iron to the stump to check the oozing which occured from innumerable points. Only one arteriole was found requiring ligation.

Upon examination of the uterus two weeks afterwards, the sound entered to a depth of three and one-half inches, turning a little toward her left side. Upon the right anterior corner of the uterus a protuberance about the size of a goose egg could be felt.

Her present general condition is good. She had an attack of menorrhagia a week after the operation, which was two weeks after previous menstrual period, and has had one every three weeks since. She is taking one-half fluid ounce each, of Squibbs' fluid extract of ergot, and extract. gosspii radicis. On examination, May 14th, the uterus seems a little smaller and menorrhagia not so profuse.

I note the following points of interest :

1. The occurrence of both tumors in the same person.

2. The slow growth of the vaginal tumor as compared with the uterine.

3. Sloughing of the capsule right after protrusion from the vagina without impairment of the vitality of the tumor proper.

4. Entire absence of sensitiveness to strong acids.

5. The production of a pedicle by ligation of the proximal end with a fine wire.

6. Sterility antedating the discovery of the uterine tumor.

7. The dating of the active growth of the tumor from the time of her husband's death.

8. The influence of ergot upon the uterine tumor.

125 State Street.

